# Fractional CO_2_ laser treatment effect on cervicovaginal lavage zinc and copper levels: a prospective cohort study

**DOI:** 10.1186/s12905-021-01379-1

**Published:** 2021-06-06

**Authors:** Attila G. Sipos, Krisztina Pákozdy, Szilvia Jäger, Kindra Larson, Peter Takacs, Bence Kozma

**Affiliations:** 1 Department of Obstetrics and Gynecology, Faculty of Medicine, University of Debrecen, 98. Nagyerdei krt., Debrecen, 4032 Hungary; 2 Division of Female Pelvic Medicine and Reconstructive Surgery, Department of Obstetrics and Gynecology, Eastern Virginia Medical School, 825 Fairfax Avenue, Suite 526, Norfolk, VA 23507-2007 USA; 3 Fempharma Ltd, Vígkedvű Mihály utca 21. 2/5., Debrecen, 4024 Hungary

**Keywords:** CO_2_ laser, Fractional laser, Vagina, Zinc, Copper, Cervicovaginal fluid

## Abstract

**Background:**

The basic principle of vaginal laser therapy is the rejuvenation of the affected tissue. Zinc and copper are essential nutritional trace elements and have a key role in connective tissue homeostasis. We aimed to investigate the effect of vaginal, fractional CO_2_ laser treatment on cervicovaginal lavage (CVL) zinc and copper levels.

**Methods:**

Twenty-nine postmenopausal women with symptoms of vaginal dryness were enrolled in our prospective cohort study. Three treatments with MonaLisa Touch CO_2_ laser system were performed four weeks apart. At each treatment CVL was collected, Vaginal Health Index (VHI) was obtained, and Visual Analog Scale (VAS) for vaginal dryness was assigned by patients. Zinc and copper concentrations were measured with optical emission spectrometry before each treatment and six weeks after the 3rd treatment.

**Results:**

The VHI scores significantly improved after each laser treatment (mean ± SD VHI score, 13.03 ± 4.49 before vs. 15.55 ± 4.35 after the 1st, 17.79 ± 4.57 after the 2nd and 19.38 ± 4.39 after the 3rd treatment, *P* < 0.01). Similarly, VAS scores reflected improvement (mean ± SD VAS score 6.59 ± 2.86 before vs. 4.17 ± 2.86 after the 1st, 2.45 ± 2.43 after the 2nd and 1.41 ± 1.94 after the 3rd treatment, P < 0.01). CVL zinc levels were significantly higher compared to copper levels (0.06 ± 0.04 vs. 0.006 ± 0.006 mg/L, P < 0.01) at baseline. While copper levels remained the same through treatments, the CVL zinc level was significantly higher after the second laser treatment compared to the baseline.

**Conclusions:**

Fractional CO_2_ laser treatment of the vagina impacts CVL zinc and copper levels differently. While CVL copper levels were not different after each laser treatment, zinc levels were significantly higher after the second treatment before returning to baseline values.

## Background

By aging, the female body inevitably becomes affected by the hormonal changes of menopause. Due to depleted ovarian function, the level of circulating estrogen decreases causing histological and structural changes in the extracellular matrix (ECM) of the vaginal tissue [1]. These changes result in an adverse effect on secretion, lubrication, pH, and content of the cervicovaginal fluid (CVF), leading to the well-known complaints of vulvovaginal atrophy (VVA) [2, 3]. VVA symptoms affect up to 40% of postmenopausal women [3].

Estrogen is the only Food and Drug Administration (FDA) approved treatment of vulvovaginal atrophy. For women who have an aversion to hormonal therapy over-the-counter (OTC) products and lubricants can be offered to relieve their symptoms. Recently several publications reported on the beneficial effect of vaginal laser therapy on vaginal mucosa in VVA, vaginal health and flora, sexual function and dyspareunia, and urinary incontinence [4–13], although the FDA has not approved vaginal laser treatment for this indication.

Zinc and copper are essential nutritional trace elements and have several structural and biochemical roles [14]. Among other biochemical functions such as cellular immunity or antioxidation, this element has a vital role in the formation of extracellular matrix (ECM) and tissue regeneration [15–17]. Vaginal biopsies of mice kept on a zinc-deficient diet showed similar histological changes such as depleted estrogen status in postmenopausal women [18]. Copper-deficient chickens produce insufficient elastic tissue, resulting in vessel malformations and rupture [19]. Takacs et al. have demonstrated that zinc has a beneficial effect on the production of extracellular components in ovariectomized rats and human vaginal smooth muscle cells [20, 21]. According to their results, 20 μM zinc sulfate significantly increased the tropoelastin production of smooth muscle cells [20].

To affect these functions, inherently the elements must be present. This availability varies with dietary differences and alimentary supply. It has been suggested that zinc and copper are absorbed primarily through the small intestine by active and passive transport mechanisms [22]. Several zinc-dependent functions are affected by zinc intake or zinc status in experimental animal models and humans as well [23].

Given the potential risks (e.g., bleeding, inflammation, and patient discomfort) and ethical considerations, vaginal tissue sampling in healthy female patients to determine zinc and copper concentration is not easily feasible. Nevertheless, the quality and quantity of the cervicovaginal fluid (CVF) accurately reflect the biochemical milieu and physiological (e.g., pregnancy, menopause) or pathological (e.g., cervical abnormalities, presence of pathogens) changes of the vagina and cervix [24].

The CVF consists of Bartholin’s and Skene’s glands secretions, exfoliated cells, transudates of blood plasma through the vaginal tissue, cervical mucus, endometrial fluid, secretions from vaginal bacterial flora, and immune cells [25]. The level of particular elements is influenced by sex hormones and varies with the menstruation cycle, pregnancy, or menopause [26]. The low cost, ease of sampling, lower risks (compared to biopsies), and ability to sample more patients result in the frequent use of CVF in clinical and preclinical studies. Of the different methods available for CVF sampling (swabs or brushes, wicks such as tampons, strips, or sponges or cups), cervicovaginal washing/lavage (CVL) seems to be reliable for further analysis [27–29]. Additionally, cervicovaginal lavage (CVL) provides an excellent opportunity to collect samples all over the female lower genitourinary tract rather than local sampling during biopsies or using swabs or strips.

Previously a significant moderate positive correlation has been found between vaginal maturation values (VMV) and CVL zinc levels. CVL zinc levels were significantly lower in women with vaginal atrophy (VMV < 50) and CVL zinc levels could be used as a marker of vaginal atrophy [30].

Our pilot study aimed to investigate the zinc and copper levels of the cervicovaginal lavage after fractional CO_2_ laser treatment. Considering zinc is required for the regeneration of vaginal tissues, we hypothesized that the laser treatment of the vagina would increase the CVL zinc levels, as a reflection of increased vaginal tissue zinc levels.

## Methods

We enrolled women into our prospective cohort study at the outpatient urogynecology clinic of the Department of Obstetrics and Gynecology, University of Debrecen, Hungary, between 6/2017 and 6/2018. Postmenopausal women with the chief complaint of vaginal dryness were asked to participate in the study. We defined postmenopausal status if patients had at least 12 continuous months of amenorrhea without any other evident reason or permanently elevated follicle-stimulating hormone (FSH) blood levels (≥ 30 mIU/mL). Exclusion criteria were pregnancy, hormone therapy (local or systemic) within the past six months, concurrent vaginal infection, cytological atypia, dysmenorrhea, POP > Stage 2, according to the pelvic organ prolapse quantification system [POP-Q] [31], severe urinary or fecal incontinence (FI) or any disease which would influence the study protocol. Also, patients were asked to refrain from vaginal intercourse for three days before and two weeks after each treatment.

At the first general gynecological visit, a medical history was taken (age, BMI, previous deliveries, menstruation cycle, the onset of menopause, hormonal therapy). The participants underwent 3 intravaginal microablative CO_2_ laser therapy 4 weeks apart and were asked to mark the severity of their symptoms for vaginal dryness on a 0–10 Visual Analogue Scale (VAS) at the following timepoints: “baseline” before the first treatment; after the 1st treatment (right before the 2nd treatment); after the 2nd treatment (right before the 3rd treatment), and six weeks after the final, 3rd treatment of CO_2_-Laser system (SmartXide2V2LR, MonaLisa Touch®, DEKA, Florence, Italy). A score of 0 indicated the absence of a symptom and a score of 10 the worst possible symptoms. The clinical evaluation was performed by a board-certified obstetrician and gynecologist blinded to the specific study-related information. The clinical data gathered included components of the Vaginal Health Index Score (VHI): elasticity, fluid secretion, pH, and epithelial mucosa integrity and moisture components. Each component is scored on a scale of 1 (worst) to 5 (best) [32]. Lower scores indicate more severe atrophy. VHI was calculated at each time point, similarly to the VAS. Demographic and pertinent clinical information was recorded prospectively and stored in a dedicated database.

Our study was approved by the Hungarian National Institutional Review Medical Research Council. All women signed written informed consent before participating in our research. There were no withdrawals or discontinuation of treatment due to adverse events.

### Laser therapy

For laser treatment, a microablative, fractional CO_2_ laser system (SmartXide2V_2_LR, Deka, Florence, Italy) was utilized, with a specific 360-degree probe designed for intravaginal procedures. Laser beams were fractionally emitted in small points (DOTs) around the vaginal mucosa during treatment. To achieve the required effect, the laser was used in D-Pulse mode. Depth was set, laser power, dwell time, and spacing were adjusted: SmartStak 1, 30 watts power, 1000 μs dwell time, and 1000 μm spacing.

### Cervicovaginal lavage

The cervicovaginal lavage fluid was collected during each visit, ensuring the absence of recent sexual activity or vaginal examination within three days. For sampling, the selected patients were in the lithotomy position, and a single-use plastic speculum was applied to open the vagina. After that, 10 ml of sterile 0.09% NaCl solution were injected into the vagina, trying to rinse as much mucosal surface as possible. The lavage fluid was introduced into the vagina with a plastic syringe for 60 s with three successive aspirations and emptying to the vaginal walls and cervix. The total amount of wash fluid is then recovered from the posterior fornix by syringe aspiration. CVL fluid was stored in plastic test tubes at − 80 °C until analysis.

### Sample pre-treatment

After volume measurement, the fluid samples of 5 ml were transferred without loss into 50 ml glass beakers by washing out the storage test tubes with 2 ml 65% (m/m) analytical pure nitric acid (Sigma-Aldrich, USA). These were then dried completely on an electric hot plate. An additional volume of 4 ml nitric acid was added to the samples with continued heating to eliminate the organic matter until dryness. After cooling back to room temperature, an additional 1.00 ml of 30% (m/m) analytical pure hydrogen peroxide (Sigma-Aldrich, USA) and 1.00 ml of ultrapure water (MilliQ, Millipore System, Merck, Germany) was added to finalize the oxidation of remaining organic materials. The resulted dried samples first were diluted with 5 ml of ultrapure water (MilliQ, Millipore System, Merck, Germany) and then transferred into volume calibrated plastic test tubes with the help of an ultrasound bath and filled up to 10.00 ml with 0.1 M nitric acid and stored at 4 °C in a refrigerator until measurement.

The purity of acids was verified by digesting blank samples containing only the chemicals but no samples. All samples were kept in polypropylene tubes at 4 °C until analysis.

### Elemental analysis

The zinc and copper concentrations of the pre-treated fluid samples were measured by inductively coupled plasma optical emission spectrometry (ICP-OES 5100, Agilent Technologies, USA). The measurements were conducted in SVDV (Synchronous Vertical Dual View) mode, gaining intensity data from the axial and radial view, simultaneously. We applied an automatic sample introduction (SPS 4, Agilent Technologies, USA), and measured the samples in a randomized design. We performed measurements to generate a five-point calibration curve for the quantitative analysis of copper and zinc. Calibration solutions were diluted from a multi-element standard of 1000 mg/L (ICP standard IV, Merck, Germany) with 0.1 M nitric acid in ultrapure water. We expressed the trace element concentration of vaginal fluid samples in mg/L. The operating parameters of ICP-OES are described in Table 1.

**Table 1:** The operating parameters of ICP-OES 5100 (Agilent Technologies)

ICP-OES operating parameters	
Repetition	3
Pump speed	15 rpm
Uptake time	20 s, fast pump
Rinse time	30 s, fast pump
Read time	10 s
RF energy	1.20 kW
Stabilization time	7 s
View mode	SVDV
view height	0 mm
Nebulizer gas	0.70 l/min
Plasma gas	12.0 l/min

### Statistical analysis

The statistical analysis was performed with SigmaStat/SPSS (SPSS Inc., Chicago, IL) software. To describe the clinical and demographic characteristics, means and standard deviations were used for continuous variables. Wilcoxon rank-sum test was used to compare the differences between the baseline scores and scores after subsequent treatments. Differences were considered significant when the P-value was less than 0.05. Data are presented as mean values (± standard deviation, SD) if not otherwise specified.

## Results

Twenty-nine postmenopausal women with the chief complaint of vaginal dryness were enrolled in our study. The average age was 58.24 ± 8.60 years, and on average 11 ± 8 years had passed since their last menstrual period. The rest of the demographic information is described in Table 2.

**Table 2:** Clinical and demographic characteristics

Variable	Mean ± SD
Number of women, N	29
Age (years)	58.24 ± 8.60
Gravida	2.38 ± 1.32
Para	1.90 ± 0.77
Last menstrual period (years)	11 ± 8
Body Mass Index (kg/m^2^)	26.40 ± 5.06

Clinical evaluation revealed no other apparent reason for the vaginal dryness other than menopausal vaginal atrophy. We have found a strong negative correlation between VAS and VHI (*r* = -0.681, *P* < 0.01).

The VHI improved significantly after each treatment compared to the baseline (mean ± SD VHI score, 13.03 ± 4.49 before vs. 15.55 ± 4.35 after the 1st, 17.79 ± 4.57 after the 2nd and 19.38 ± 4.39 after the 3rd treatment, *P* < 0.01, Table 3). The patient-reported VAS vaginal dryness score was significantly lower after each laser treatment (mean ± SD VAS score, 6.59 ± 2.86 before vs. 4.17 ± 2.86 after the 1st, 2.45 ± 2.43 after the 2nd and 1.41 ± 1.94 after the 3rd treatment, P < 0.01, Table 3).

**Table 3:** Vaginal Health Index (VHI) and Visual Analog Scale (VAS) for vaginal dryness before and 4 weeks after the 1st, 2nd, and 3rd treatment with CO_2_ vaginal laser

	CO_2_ laser treatment				P-value		
	Baseline	After 1st	After 2nd	After 3rd	Baseline versus 1st	Baseline versus 2nd	Baseline versus 3rd
VHI	13.03 ± 4.49	15.55 ± 4.35	17.79 ± 4.57	19.38 ± 4.39	< 0.01	< 0.01	< 0.01
VAS	6.59 ± 2.86	4.17 ± 3.02	2.45 ± 2.43	1.41 ± 1.94	< 0.01	< 0.01	< 0.01

CVL zinc levels were significantly higher compared to copper levels at baseline (mean ± SD, mg/L, 0.06 ± 0.04 vs. 0.006 ± 0.006, P < 0.01). The first laser treatment had no significant effect on the CVL zinc levels (Table 4). After the second laser treatment, CVL zinc levels were significantly higher, but after the 3rd treatment, CVL zinc levels returned to the baseline values. Contrary to zinc levels, copper levels in the CVL of women undergoing vaginal CO_2_ laser remained similar after three vaginal laser treatments (Table 4).

**Table 4:** Cervicovaginal lavage zinc and copper levels before and 4 weeks after the 1st, 2nd, and 3rd treatment with CO_2_ vaginal laser

	CO_2_ laser treatment				P-value		
	Baseline	After 1st	After 2nd	After 3rd	Baseline versus 1st	Baseline versus 2nd	Baseline versus 3rd
Zinc (mg/L)	0.06 ± 0.04	0.07 ± 0.05	0.10 ± 0.09	0.07 ± 0.07	NS	< 0.01	NS
Copper (mg/L)	0.006 ± 0.006	0.006 ± 0.008	0.006 ± 0.007	0.005 ± 0.005	NS	NS	NS

## Discussion

To our knowledge, we are the first to investigate zinc and copper levels in the cervicovaginal lavage (CVL) after CO_2_ laser treatment. Fractional CO_2_ laser treatment of the vagina affected CVL zinc and copper levels differently. While CVL copper levels were not different after each laser treatment, zinc levels were significantly higher after the second treatment before returning to baseline values after the third laser treatment.

There are numerous publications available in the literature describing the impact of the fractional CO_2_ laser on the vaginal environment. Besides physiological changes, the unfavorable effect of radiation used to treat cervical cancer also influences vaginal health [33, 34]. The use of laser treatment to revert the adverse effect of radical surgeries or radiotherapy for gynecological malignancy is also under active investigation [35]. The number of publications still rising, although the FDA has not approved vaginal laser treatment for those indications. Zerbinati et al. demonstrated an increased number of active fibroblasts, glycogen-rich cells, and augmented content of extracellular matrix (ECM) elements such as elastin and collagen [36] in vaginal mucosa after laser treatment. Salvatore et al. described similar changes in the vaginal tissues in response to treatment [37]. Other authors investigating postmenopausal vaginal cytology after laser treatment found significant improvement in vaginal maturation values (VMV) and/or vaginal symptoms, which inversely related to atrophy [7, 38, 39]. Athanasiou’s study revealed that this form of therapy (laser) helps repopulate existing bacteria and restore normal premenopausal flora in the vagina [12]. Besides, publications are reporting the beneficial effect of vaginal CO_2_ laser on vulvodynia (“pain in the vulva”) and lichen sclerosis as well [40, 41].

The basic principle of its remodeling effect is that the laser therapy energy is absorbed by water in the treated tissue causing a cascade of events to happen [37]. The CO_2_ laser beams are delivered fractionally, causing ablative micro-millimeter thermal damages. As a result, a rapid epithelial repair mechanism begins. In the short term, the collagen fibers became thicker and shorter. After a while, neovascularization, increased fibroblast activity, and newly formed collagen fibers are detectable in the epithelium [36, 37]. Previous studies illustrated the critical role of Zn supply in connective tissue formation [15, 16]. Based on these findings, we could presume that the collagen genesis and vaginal ECM remodeling induced by the thermal effect of CO_2_ laser occur more effectively in a zinc-rich environment.

The zinc and copper balance in the vagina of pre-and postmenopausal women is still under investigation. Based on results from animal experiments, it is known that among other biological functions like cellular immunity or antioxidation, zinc plays a vital role in ECM formation and wound healing [15]. The plasma zinc level of buffalos with antepartum vaginal prolapse was found to be significantly lower compared to healthy pregnant buffalos [42]. Vaginal samples of mice kept on a zinc-deficient diet showed similar histological changes to depleted estrogen status in postmenopausal women. The zinc tissue level in the uterus is also the lowest after menopause [18]. Takacs et al. have demonstrated that zinc has a beneficial effect on the production of extracellular components in ovariectomized rats and human vaginal smooth muscle cells as well [20, 21].

Copper also plays an important role in connective tissue biosynthesis and physiology. Animal experiments on copper-deficient chicks and swines revealed histological evidence of abnormal elastic tissue in the aorta resulting in major vessel rupture [19]. Rucker et al. demonstrated that copper deficiency resulted in decreased mechanical strength in tissues high in elastin and collagen (blood vessels, tendons, and bone) due to insufficient collagen and elastin cross-linking [43, 44].

Although vaginal wall biopsies would be a more direct way to gather more information about the mechanisms maintaining the vaginal zinc and copper balance, the in vivo application of this invasive method raises ethical questions and limits specimen collection. Previous studies revealed that the cervicovaginal lavage (CVL) is a useful way to collect a sample from the female lower genitourinary tract [27–29]. The content of the cervicovaginal lavage fluid (CVL) accurately reflects the physiological changes of the vagina and cervix during pregnancy or menopause and can detect pathogens, cervicopathological changes, and the presence or absence of different proteins and minerals during several genital diseases [29, 45, 46]. This suggests it could be a useful surrogate for vaginal biopsy.

Though the exact pathways and mechanisms behind the zinc and copper transport from the vaginal epithelial tissue to the cervicovaginal fluid (CVF) are still under investigation, there is likely a correlation between the level of these elements in vaginal tissue and cervicovaginal lavage (CVL). Based upon this assumption, we conclude that the extracellular matrix (ECM) regeneration and repair mechanisms in the vaginal epithelium induced by the CO_2_ laser treatment require increased tissue zinc concentration, and the elevated CVL zinc concentration detectable after CO_2_ laser therapy reflects this increased need for zinc.

We believe that the strength of our study is that this is a new perspective, bringing novel information to the existing literature. The primary weaknesses of our study are the relatively small sample size and lack of a control group. A future trial designed with a laser treatment arm and a sham treatment arm would provide more critical data. Besides, full-thickness vaginal wall biopsies rather than CVL as a surrogate marker would give conclusive results on vagina tissue levels of zinc and copper in response to treatment.

## Conclusion

Fractional CO_2_ laser treatment of the vagina significantly improved the symptoms of vaginal dryness in postmenopausal women. In addition to subjective improvement, the VHI has improved significantly as well. Laser treatment affected the levels of zinc and copper in CVL differently. While CVL copper levels were not different after each laser treatment, zinc levels were significantly higher after the second treatment before returning to baseline values. The fact that zinc and not copper levels changed in the CVL, suggests that zinc may play a stronger role in the remodeling process observed with laser treatment. Further studies are required to explore zinc's role in the CVL.

## Data Availability

All data used and/or analyzed during the current study are available from the corresponding author on reasonable request.

## Abbreviations

CVL: Cervicovaginal lavage
CVF: Cervicovaginal fluid
VAS: Visual analog scale
VHI: Vaginal health index
ECM: Extracellular matrix
FDA: United States Food and Drug Administration
OTC: Over-the-counter
VVA: Vulvovaginal atrophy
VMV: Vaginal maturation value
FSH: Follicle-stimulating hormone
POP: Pelvic organ prolapses
POP-Q: Pelvic organ prolapse quantification system
UI: Urinary fecal incontinence
FI: Fecal incontinence
BMI: Body mass index
SVDV: Synchronous Vertical Dual View
USA: United States of America
